# Retraction of: ‘Reef fishes can recognize bleached habitat during settlement: sea anemone bleaching alters anemonefish host selection’ (2016) by Scott and Dixson

**DOI:** 10.1098/rspb.2023.1346

**Published:** 2023-06-28

**Authors:** Proceedings B Editorial team

*Proc. R. Soc. B* **283**, 20152694. (Published online 25 May 2016). (https://dx.doi.org/10.1098/rspb.2015.2694)

*Proceedings B* is committed to the integrity of the scientific record. It therefore takes allegations concerning the validity of research published in the journal extremely seriously. At same time, however, it is conscious of the potentially far-reaching consequences of these allegations for authors and for the institutions in which they work. We therefore aim to take a fair and balanced approach to investigations on the reliability of research by thoroughly assessing the combined weight of evidence that is made available to us at any given time when deciding if an article requires a correction or a retraction. Although assessments may be informed by investigations by other journals and/or institutions, ultimately *Proceedings B* only investigates and adjudicates articles that it has published.

When *Proceedings B* was first made aware of concerns regarding an article by Scott & Dixson published in 2016 [1], it established a team of editors to launch its own investigation of the issues raised. This resulted in the publication of a correction by the authors that responded to those concerns [2]. Furthermore, it also resulted in making the accompanying data publicly available. The publication of the correction on 8 July 2022 as well as the data on which the article was based then enabled further scrutiny by the scientific community.

Subsequently, a new set of allegations surfaced; some submitted to the journal and others made in the public domain. The team of editors was tasked with further assessment of the combined weight of this body of evidence, including reports from two external experts on flume experiments. We conducted a detailed quantitative assessment of the Scott & Dixson data as well as analyses provided to us by several whistle blowers. Our conclusion at that time was that there was insufficient evidence to warrant retraction [3]. Although the journal received a request for retraction from the University of Delaware (Dr Dixson's institution) based on their own internal investigation, the University did not share their evidence with the journal. The fact that no new evidence was provided to us made it impossible to comply with the University of Delaware's request.

Recently, Southern Cross University, the employer of the first and corresponding author Dr Anna Scott advised us of substantial doubts regarding the new timelines stated in the correction and the feasibility of the experimental design. They requested that we retract the Scott and Dixson (2016) article as well as its correction. Therefore, having considered the full body of evidence, including these new disclosures, *Proceedings B* has now lost confidence in the reliability of the data that provide the basis for the main findings and conclusions in Scott and Dixson (2016) and has therefore decided to retract the article and the associated correction.
